# PCSK9 Inhibition Reduces Depressive like Behavior in CUMS-Exposed Rats: Highlights on HMGB1/RAGE/TLR4 Pathway, NLRP3 Inflammasome Complex and IDO-1

**DOI:** 10.1007/s11481-023-10060-3

**Published:** 2023-02-13

**Authors:** Nevien Hendawy, Tala H. Salaheldin, Sally A. Abuelezz

**Affiliations:** 1 Clinical Pharmacology Department, Faculty of Medicine, Ain-Shams University, Cairo, Egypt; 2 Student at Faculty of Medicine, Cairo University, Cairo, Egypt

**Keywords:** Depression, PCSK9, HMGB1/RAGE/TLR4 pathway, NLRP3 inflammasome, Indoleamine2,3-dioxygenase-1

## Abstract

Ample evidence has pointed to a close link between cardiovascular diseases (CVD) and depression. Inflammatory pathways including the high-mobility-group-box-1 protein, receptor-for-advanced-glycation-end-products and toll-like-receptor-4 (HMGB1/RAGE/TLR4) and nucleotide-binding domain (NOD)–like receptor protein 3 (NLRP3) inflammasome pathways are thought to be crucial players in this link. Activation of these pathways ends by releasing of different inflammatory mediators involved in CVD and depression pathophysiology. In the brain, this inflammatory process enhanced indoleamine2,3-dioxygenase-1 (IDO-1) activation with subsequent alteration in kynurenine/tryptophan levels causing depression. Based on the favorable anti-inflammatory effects of Alirocumab, the proprotein-convertase-subtilisin/kexin-type-9 (PCSK9) inhibitor, used in different CVD, this study was designed to investigate its potential antidepressant effect. The behavioral and neurochemical effects of concomitant treatment of Alirocumab at doses of (4, 8 and 16 mg/kg/week subcutaneously) in Wistar rats exposed to chronic unpredictable mild stress (CUMS) for 6 weeks were assayed. Alirocumab prevented CUMS-induced depressive-like-behaviors exhibited in open-field and forced-swimming tests, and hypothalamus–pituitary–adrenal axis hyperactivity (adrenal gland weight and serum corticosterone). Alirocumab prevented CUMS-induced alteration in hippocampal kynurenine/tryptophan levels and pro-inflammatory cytokines tumor-necrosis-factor-alpha, interleukin-1beta (IL-1β), IL-2 and IL-6. Western blot and PCR analysis showed that Alirocumab favorably modulated the HMGB1/RAGE/TLR4 axis, nuclear-factor-kappa-beta, NLRP3 inflammasome complex and IDO-1 in the hippocampus of CUMS rats. These effects were correlated to the level of PCSK9 expression. The behavioral and biochemical findings indicated the potential antidepressant effect of PCSK9 inhibition by Alirocumab.

**Graphical Abstract:**

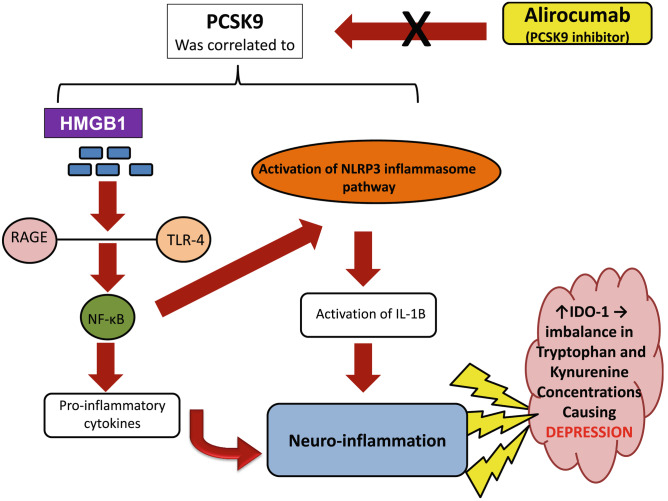

## Introduction

Proprotein convertase subtilisin/kexin type 9 (PCSK9) is a protease which was primarily associated with lipoprotein homeostasis. The role of PCSK9 in cholesterol metabolism was initially identified in families with history of familial hypercholesteremia as it targets low-density lipoprotein (LDL)-cholesterol in the blood. It acts by binding to the LDL-receptors causing its degradation with subsequent elevation of LDL-cholesterol levels. Accordingly, PCSK9 inhibition had opened a new avenue in the management of dyslipidemia. PCSK9 inhibitors are now one of the emerging non-statin lipid lowering agents which exhibited an increasing clinical benefits in patients and are used in different cardiovascular diseases (CVD) especially coronary heart diseases (CHD), myocardial infarction (MI), and stroke (Joseph and Robinson 2015; Tsivgoulis et al. 2018).

CVD are well documented as a leading cause of morbidity and mortality worldwide. Decades of research has revealed links between CVD and neuropsychiatric disorders. A higher prevalence of depression in CVD patients has been demonstrated where studies showed that almost 20% of all patients with CHD are suffering from depression. Depression also represents the most common and serious comorbidity of stroke, with nearly one third of stroke survivors develop post stroke depression which exacerbate the deterioration of physical, functional and cognitive recovery, as well as enhanced levels of mortality (De Hert et al. 2018; GBD 2016 Stroke Collaborators 2019; Mohammad et al. 2019; Kotov et al. 2020).

Recently, studying the biological underpinnings of the intriguing relationship between depression and CVD had addressed inflammation as a significant contributor in this relationship. Inflammation has been demonstrated through different signaling pathways including the high-mobility-group-box-1 (HMGB1) protein and nucleotide-binding domain (NOD)–like receptor protein 3 (NLRP3) inflammasome pathways. HMGB1 is a chemotactic and pro-inflammatory mediator, which is recognized as a key initiator for inflammation. It acts by direct binding to the receptor for advanced-glycation-endproducts (RAGE) in addition to the toll-like receptor (TLR)-4, with subsequent activation of nuclear factor kappa beta (NF-kB) pathway releasing different pro-inflammatory cytokines as tumor necrosis-factor-alpha (TNF-α), interleukin-1beta (IL-1β) and IL-6. NLRP3 inflammasome is a part of an intracellular polyprotein complex which is up-regulated under the influence of NF-kB. It plays an important role in inflammation and is involved in the formation of the mature form of IL-1β. Activation of the aforementioned pathways with subsequent release of different inflammatory mediators are crucial players in both CVD and depression pathophysiology. In the brain, this inflammatory process enhanced indoleamine2,3-dioxygenase-1 (IDO-1) activation with subsequent alteration in kynurenine/tryptophan levels, which can be one of the main causes of the development of depression in CVD patients (Raucci et al. 2019; Wang et al. 2019; Zhang et al. 2019a; Tong et al. 2020; Wang et al. 2020; Xu et al. 2020; Wahid et al. 2021). These observations raise the attention to introduce new potential targets for the CVD therapeutic interventions where using these agents was pointed to as a promising component in preventing subsequent neuropsychiatric comorbidities.

Although PCSK9 inhibitors role was initially conceptualized as reduction of LDL-cholesterol and used in CVD, yet studies exploring their alternative pharmacological effects have pointed to their anti-inflammatory abilities in different organs and to their critical role in the brain especially through the HMGB/RAGE/TLR4 axis and NF-kB (Dwivedi et al. 2016; Filippatos et al. 2018; Lohoff 2018; Seidah et al. 2018; Abuelezz and Hendawy 2021). Accordingly, this study was designed to evaluate the ability of the PCSK9-inhibitor alirocumab (Aliro) to alleviate depressive-like-behaviors as a common comorbidity to CVD. To the authors' knowledge, studies exploring the effects of PCSK9-inhibitors on depressive–like-behaviors and trying to elucidate the underlying pathological mechanisms in rats have not been conducted hitherto.

## Material and Methods

### Animals

Sixty adult male Wistar rats weighing 150–200 g were used in the study. Animals were purchased from the Egyptian Organization for Biological Products and Vaccines VACSERA, Egypt. They were acclimated for 7 days before experimentation and kept under controlled environmental conditions; a 12-h dark/light cycle, temperature ~ 25 °C and with standard diet (Meladco for Animal Food, Egypt) and water during the experiments. All efforts were made to minimize the number and suffering of animals and the study procedures were done following the European community guidelines for care and use of experimental animals (EEC Directive of 1986) and it was approved by the Ethical Committee, Faculty of Medicine, Ain Shams University.

### Treatments and Experimental Design

Alirocumab (Sanofi,Egypt) was administrated subcutaneously (s.c) in three doses (4, 8 and 16 mg/kg/week representing Aliro-L, Aliro-M, Aliro-H respectively). Doses of alirocumab were chosen guided by human equivalent dose calculation and based on previous study (Shin and Seol 2010; Abuelezz and Hendawy 2021). Animals were randomly divided into five equal groups: the first unstressed group served as control, the second group was exposed to chronic unpredictable mild stress protocol (CUMS), daily for 6 weeks, whereas the last three groups were exposed to CUMS and concomitantly received once daily Alirocumab in a dose dependent fashion (Aliro-L, Aliro-M, Aliro-H) for the 6 weeks of the daily stress protocol.

A pilot study was performed to detect the effect of the drug on the behavioral tests and lipid profile in animals before the start of our study and it revealed a non-significant difference between untreated and treated normal animals.

### Chronic Unpredictable Mild Stress Protocol (CUMS)

**Fig. 1:**
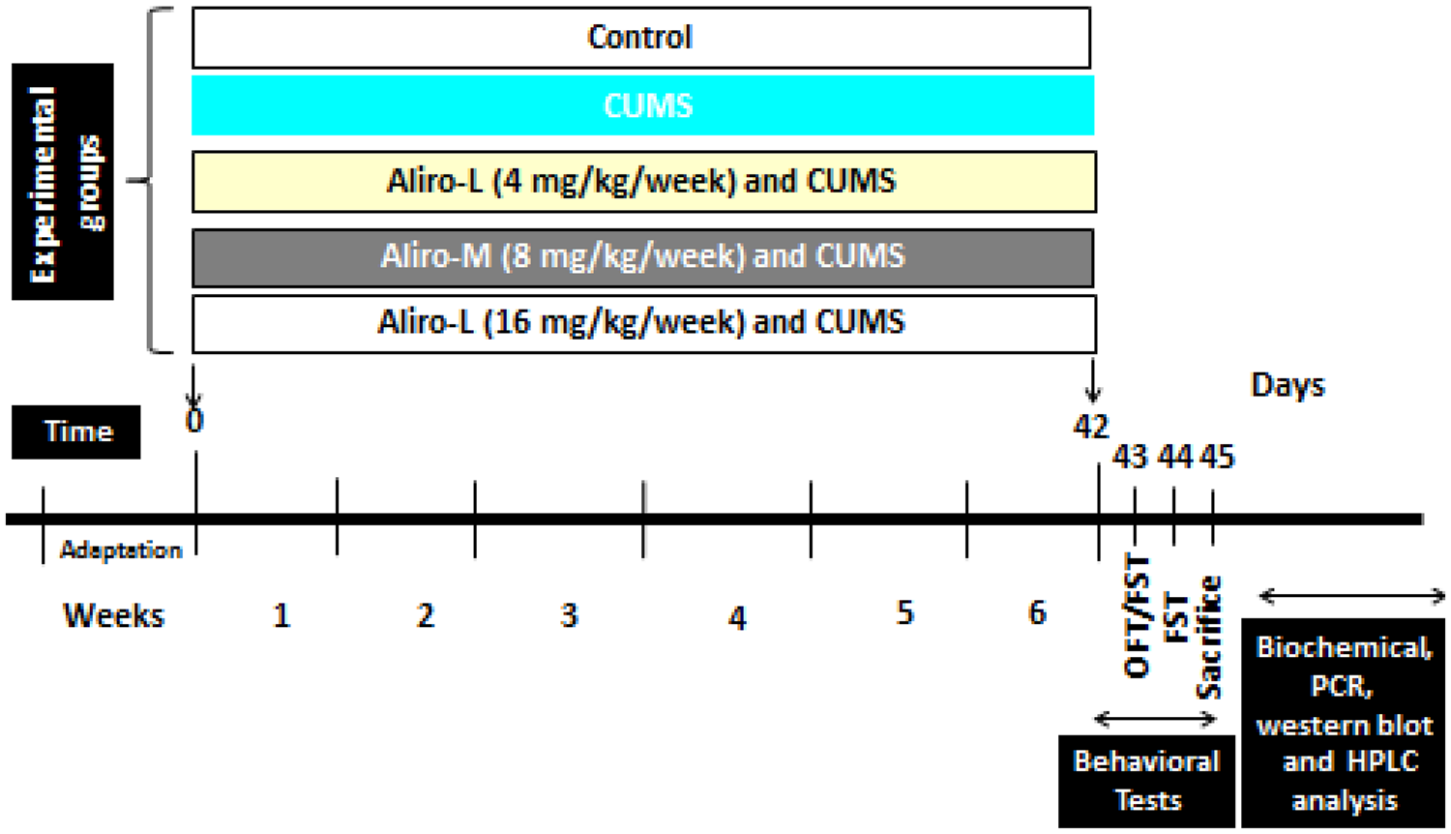
Timeline for chronic unpredictable mild stress (CUMS) and Alirocumab (Aliro) administration schedule, behavioral tests, sacrifice, biochemical, Real-time reverse transcriptase (PCR), western blot and high-performance liquid chromatography (HPLC) analysis. (OFT) open field test and (FST) forced swim test

The CUMS protocol was performed following that previously described by Abuelezz et al. (2017). Accordingly, rats were exposed to mild stressor episodes (2–3 stressors) daily for 6 weeks. The stressors are reversed light cycle, pairing, cage tilting, stroboscopic light (60 flashes/min), cold temperature (10 °C), 120 min restricted access to food (3 pellets), 60 min empty water bottles, foreign body in the cage, soiling of cage with 50–100 ml water, 60 min immobilization stress and 60 min cage agitation (cages were rotated by gentle rotation ~ 10 rpm). The stressors were applied in a semi random sequence to be unpredictable. The timeline of the experimental design and measurements are shown in Fig. 1.

### Behavioral Tests

#### Open Field Test

OFT was used to detect exploration, locomotors activity and, anxiety-related behaviors. For this purpose, a quadrangular arena 60 × 60 × 45 cm divided into 16 equal squares was used. Before starting the experiment, animals were allowed to acclimatize to the test room for 1 h, and then for 5 min each rat was individually placed in the center of the well-illuminated quadrangular arena. The number of crossed squares (visited with all 4 ft), number of entries to the central zone (central 4 squares), latency to leave the central zone and time stayed in the central zone, duration (sec) of both rearing (standing upright on the hind paws) and of grooming (face rubbing and licking both paws and fur licking) mean velocity (cm/s) and total distance travelled (cm) were all recorded. The test arena was cleaned by 70% alcohol after each rat (Abuelezz et al. 2017).

#### Forced Swimming Test

A vertical glass cylinder (diameter 22.5 cm X height 50 cm) filled with fresh water 35 cm high maintained at ≈25 °C was used for this test. For training, each rat was forced to swim individually for 15 min after the OFT. A re-exposure to the FST was done 24 h later for 5 min and the experiments were videotaped for later scoring of the immobility, swimming, and struggling time. Depressive like behavior was exhibited in the form of reduction in the time spent in active behavior (i.e. struggling or swimming) and increased the immobility time (Abuelezz et al. 2017).

Then animals were anesthetized with an intraperitoneal injection of urethane at the dose of 1.3 g/kg (Guo et al. 2007). Then rats were sacrificed by decapitation, and their brains were dissected out and washed by cold saline. The procedure was done between 10:00–12:00 am to avoid fluctuations in the glucocorticoid hormone levels.

### Adrenal Weight

Both adrenal glands were excised and weighed. The increase in adrenal gland weight is considered as an indirect indicator of the hypothalamic-pituitary adrenal (HPA) axis activation which is evident in chronic stress status due to the release of glucocorticoid hormones by the adrenals in response to the different psychological and physical stressors (Rezin et al. 2008).

### Sample Collection and Biochemical Measurements

Blood samples were collected and centrifuged for 15 min at 1000 g to separate serum. Brains were dissected and the hippocampi were quickly isolated on an ice cold plate. For each group, the right hippocampus of 6 rats was used to pro-inflammatory cytokines and the left hippocampus was used for the real-time reverse transcriptase polymerase chain reaction (PCR) analysis. For the remaining rats, the right hippocampus was used for neurotransmitter analysis and the left hippocampus was used for western blot analysis. The samples were immediately stored at − 80 °C until assayed.

#### Determination of Serum Corticosterone

Rat corticosterone enzyme-linked immune sorbent assay (ELISA) kit (DRG Instruments Gmbh, Marburg, Germany) was used according to the manufacturer’s instructions to determine serum corticosterone level.

#### Determination of Hippocampal Pro-inflammatory Cytokines

Following the instructions provided by the Millipore multiplex Rat Cytokine enzyme-linked immune sorbent assay (ELISA) Kit; IL-1β, IL-2, IL-6 and TNF-α levels were measured. Briefly, hippocampi were homogenized in 0.01 M Tris hydrochloride formed of 5.8% sodium chloride, 10% glycerol, 1% Nonidet P40 (NP-40), 0.4% of ethylenediamine tetraacetic acid (EDTA) and protease inhibitors (Complete Protease Inhibitor Cocktail Tablets, Roche). Samples were then centrifuged for 5 min at 10,000 g at 4 °C and concentrations were determined on the Luminex® platform.

#### Real-Time Reverse Transcriptase (PCR)

According to the manufacturer’s protocol, the total RNA was extracted from the hippocampi samples which were homogenized with Trizol reagent (Invitrogen, CA, USA). The cDNA was synthesized from total RNA using the high-capacity cDNA reverse transcription kit (Applied Biosystems, USA) and was amplified by the SYBR green Master Mix kit (Invitrogen, CA, USA) through comparative quantitative real-time reverse transcriptase PCR. The following primers were used: β-actin (forward) 5′-GCAGGAGTACGATGA GTCCG- 3′ and (reverse) 5′-ACGCAGCTCAGTAACAGTCC-3′; NF-κB: (forward) 5′—GCGCATCC GACCAACAATAAC-3′ and (reverse) 5′-GCCGAAGCTGCATGGACACT- 3′; TLR4 (forward) 5′—AGCTTTGGTCA GTTGGCTCT-3′ and (reverse) 5′-CAGGATGACACCATTG AAGC-3′; PCSK9 (forward) 5′-GCTTCAGCGGCTTGTTCCT-3′ and (reverse) 5′-TGCTCCTCCAC TCTCCACATAA-3′; IDO-1, (forward) 5′-AGC ACT GGA GAA GGC ACT GT-3′ and (reverse) 5′-ACG TGG AAA AAG GTG TCT GG- 3′. The mRNA expression of the tested genes was normalized to those of β-actin.

#### High-Performance Liquid Chromatography

Following the methodologies described previously by Bellac et al. (2006) a Luna high-performance liquid chromatography (HPLC) column [5 μm C18 (2) 150 × 4.60 mm; Phenomenex, Torrance, CA, USA] was used for Kynurenine determinations in hippocampal tissue homogenates. The mobile phase for ultraviolet detection consisted of; 15 mmol/L sodium acetate/acetic acid solution containing 2.7% (v/v) acetonitrile, pH 3.6. The flow rate was set at 1.3 mL/minute. The injected sample volume was 100 μL. Peaks were detected at 365 nm wavelength. 3-Nitro-ltyrosine was used as an internal standard. For Tryptophan levels determination in the hippocampal tissue homogenates, the mobile phase for the electrochemical detector HP 1049A (Hewlett Packard, Palo Alto, CA, USA) consisted of acetonitrile, 100 mM acetic acid, 100 mM ammonium acetate (10:90) v/v, and 50 mg/L ethylene diamine tetra acetic acid, pH 5.1.31 3-Methoxy-4- hydroxyphenethyl alcohol as an internal standard was used. Measurements were done at an electrode potential of 0.850 mV.

#### Western Blot Analysis

By using ice-cold RIPA buffer the total protein was isolated from hippocampal tissues. BCA Protein Assay Kit (Thermo Fisher Scientific, USA) was used to measure the protein concentrations. A nitrocellulose membrane (Millipore Co., USA) was used for immuno-blotting where the protein samples (30 μg) were separated using SDS–polyacrylamide gel electrophoresis and then transferred to. For 1 h the membrane was blocked with 5% skim milk in Trisbuffered saline (150 mM NaCl, 0.1% Tween 20, 20 mM Tris, pH 7.4). Then the proteins were detected by incubation with primary antibodies against HMGB1, RAGE at 4 °C overnight. Later the membrane was washed for 3 times in Tween 20 + PBS and was incubated by the horseradish peroxidase-conjugated secondary antibodies for 2 h. The immuno-blots were visualized by a Millipore ECL Western Blotting Detection System and the variables protein expression levels were normalized to those of β-actin.

The following antibodies were used: anti-β actin (1:2,000, sc-130657, Santa Cruz Biotechnology), anti-HMGB1 (1:1,000, ab18256; Abcam), anti-RAGE antibody (1:2,000, sc8230; Santa Cruz), anti-NLRP3 antibody (1:2,000, sc-66846, Santa Cruz Biotechnology), anti-ASC antibody (1:2,000, sc-22514-R, Santa Cruz Biotechnology), pro and cleaved Caspase1 antibody (1:2,000, sc-514, Santa Cruz Biotechnology) and pro and mature IL-1β (1:1,000, AF-401-NA, R&D Systems).

### Statistical Analysis

Data were expressed as mean ± standard error of the mean (SEM). Using Graph-pad prism 5 statistical comparisons were carried out using one-way analysis of variance (ANOVA) followed by post-hoc Bonferroni test. The minimal level of significance was identified at P < 0.05. Correlation-coefficient analysis was performed by the Pearson's r.

## Results

### Effect of Alirocumab on CUMS-Induced Behavioral Changes in Rats

#### Open Field Test

**Fig. 2:**
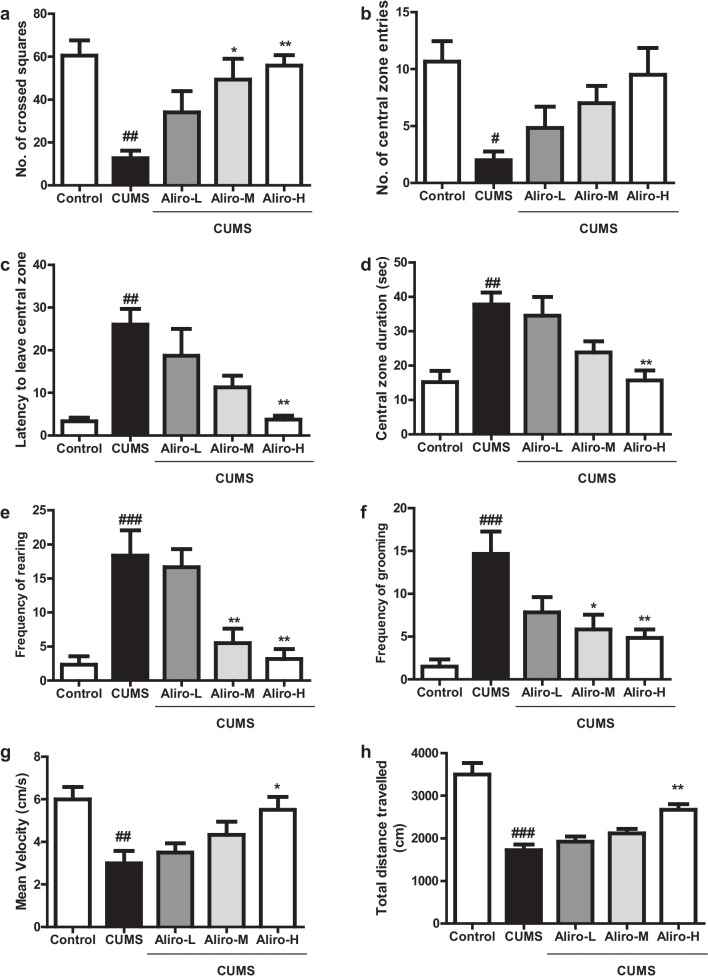

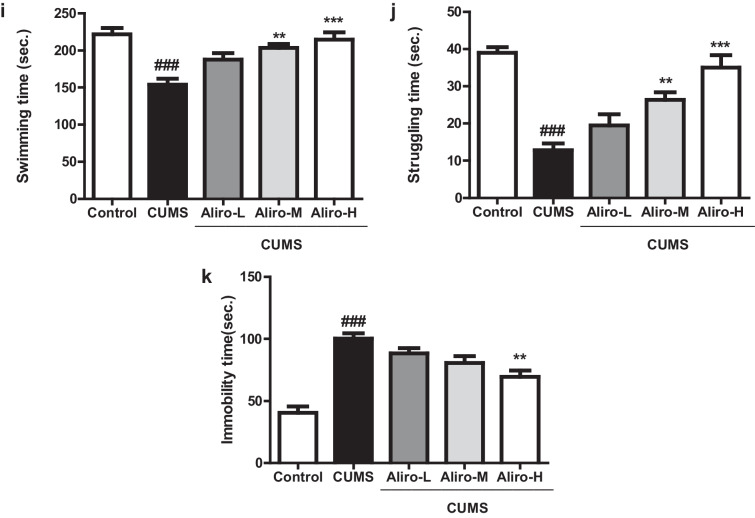
Effect of alirocumab (Aliro-L,Aliro-M and Aliro-H) on CUMS-induced behavioral Changes in rats in: **a**–**h** open-field test and **i–k** Forced swimming test; **a** Number of total crossed squares, **b** Number of central zone entries, **c** Latency to leave the central zone, **d** Central zone duration, **e** Frequency of rearing, **f** Frequency of grooming, **g** Mean velocity, **h** Total distance travelled, **i** Swimming time, **j** Struggling, **k** Immobility time. ^#^*P* < 0.05, ^##^*P* < 0.01, ^###^*P* < 0.001 vs. control group and ^*^P < 0.05, ^**^P < 0.01 vs. CUMS group; by one-way ANOVA followed by Bonferroni post-hoc test

As depicted in Fig. 2a–h, CUMS protocol led to a significant (*P* < 0.01) decrease in the number of crossed squares (F_(4,55)_ = 6.76, *P* < 0.01), significant (*P* < 0.05) decrease in number of central zone entries (F_(4,55)_ = 4.03, *P* < 0.05), significant *(P* < 0.01) increase in central zone duration (F_(4,55)_ = 7.63, *P* < 0.01), significant (*P* < 0.01) increase in latency to leave central zone (F_(4,55)_ = 7.71, *P* < 0.01), significant (*P* < 0.001) increase in frequency of rearing (F_(4,55)_ = 10.14, *P* < 0.001), a significant (*P* < 0.001) increase in frequency of grooming (F_(4,55)_ = 8.17, *P* < 0.001), a significant (*P* < 0.01) decrease in mean velocity (F_(4,55)_ = 5.06, *P* < 0.01), and a significant (*P* < 0.001) decrease in total distance travelled (F_(4,55)_ = 19.22, *P* < 0.001) compared to control group. Alir-M produced a significant (*P* < 0.05) increase in the number of crossed squares, and significant decrease in frequency of rearing and grooming (*P* < 0.01, *P* < 0.05 respectively). Aliro-H significantly increased the number of crossed squares, the mean velocity and total distance travelled (*P* < 0.01, *P* < 0.05, *P* < 0.01 respectively), decreased latency to leave central zone, central zone duration, frequency of rearing and grooming (*P* < 0.01, P < 0.01, *P* < 0.01, *P* < 0.01 and *P* < 0.01) respectively compared to CUMS group.

#### Forced Swimming Test

As exhibited in Fig. 2i–k, CUMS protocol for 6 weeks led to behavior despair in rats indicated by significant (*P* < 0.001) decrease in swimming time (F_(4,55)_ = 10.72, *P* < 0.001) and struggling time (F_(4,55)_ = 19.10, *P* < 0.001) and a significant (P < 0.001) increase in immobility time (F_(4,55)_ = 21.09, P < 0.001) compared to control group. Aliro-M and Aliro-H significantly increased swimming time and struggling (*P* < 0.01, *P* < 0.001 respectively) and Aliro-H significantly decreased immobility time (*P* < 0.01 relative to CUMS group.

### Effect of Alirocumab on CUMS-Induced Changes in Serum Corticosterone Level and Adrenal Glands Weight in Rats

**Fig. 3:**
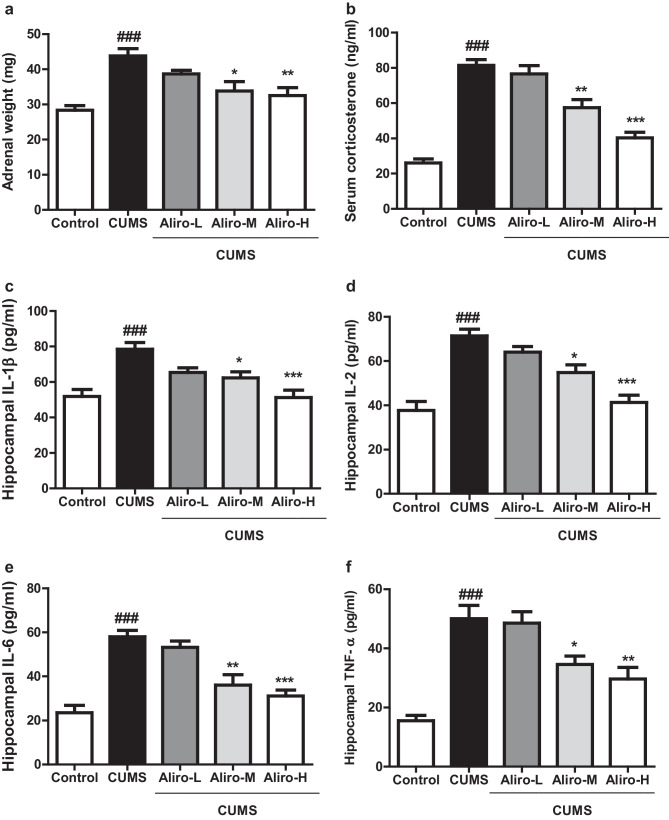
Effect of alirocumab (Aliro-L,Aliro-M and Aliro-H) on CUMS-induced Changes in **a**, **b** adrenal glands weight in rats and serum corticosterone level, **c**–**f** Hippocampal pro-inflammatory cytokines; **a** Adrenal glands weight, **b** Serum corticosterone level Hippocampal pro-inflammatory cytokines; **c** IL-1β, **d** IL-2, **e** IL-6, and **f** TNF-α levels. ^###^*P* < 0.001 vs. control group and ^*^P < 0.05, ^**^P < 0.01, ^***^P < 0.001 vs. CUMS group; by one-way ANOVA followed by Bonferroni post-hoc test

As shown in Fig. 3a, b, CUMS protocol for 6 weeks in Wistar rats induced significant increase in adrenal glands weight (F_(4,55)_ = 9.69, *P* < 0.001) and a significant increase in serum corticosterone level (F_(4,55)_ = 39.98, *P* < 0.001) compared to control group. Both Aliro-M and Aliro-H were able to prevent the elevation in both adrenal gland weight (*P* < 0.05 and *P* < 0.01 respectively) and serum corticosterone level (*P* < 0.05 and *P* < 0.001 respectively) compared to the CUMS group.

### Effect of Alirocumab on CUMS-Induced Changes in Hippocampal Pro-inflammatory Cytokines in Rats

As exhibited in Fig. 3c–f, a significant (*P* < 0.001) elevation in hippocampal levels of IL-1β (F _(4,25)_ = 9.34, *P* < 0.001), IL-2 (F_(4,25)_ = 18.44, *P* < 0.001), IL-6 (F _(4,25)_ = 18.51, *P* < 0.001) and TNF-α (F_(4,25)_ = 16.31, *P* < 0.001) in rats subjected to CUMS group compared to control group. Aliro-M and Aliro-H were able to significantly (P < 0.05, P < 0.001, respectively) decrease the hippocampal IL-1β and IL-2. Aliro-M and Aliro-H significantly reduced the hippocampal IL-6 (*P* < 0.01, *P* < 0.001 respectively) and TNF-α (*P* < 0.05, *P* < 0.01 respectively) in contrast to CUMS group.

### Effect of Alirocumab on CUMS-Induced Changes in Hippocampal PCSK9, NF-κB, TLR-4 and Indolamine 2, 3- Dioxygenase 1 (IDO-1) mRNA Expression in Rats

**Fig. 4:**
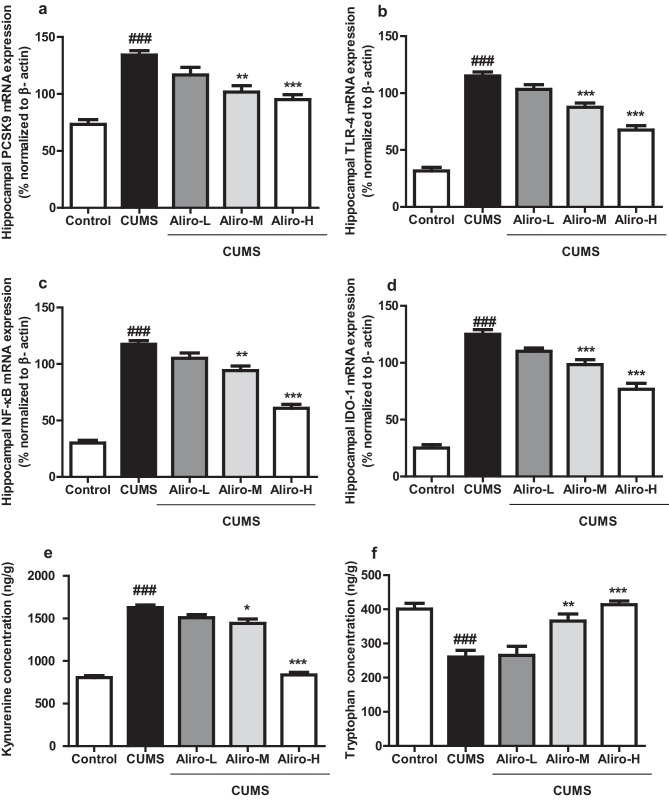
Effect of alirocumab (Aliro-L,Aliro-M and Aliro-H) on CUMS-induced Changes in **a–d** Hippocampal PCSK9, TLR-4, NF-κB, and Indolamine 2, 3-Dioxygenase 1 (IDO-1) mRNA expression **e**, **f** Hippocampal Kynurenine and Tryptophan concentrations; **a** PCSK9, **b** TLR-4,, **c** NF-κB, and **d** IDO-1 levels, **e** Kynurenine concentration, **f** Tryptophan concentration.^###^*P* < 0.001 vs. control group and ^*^P < 0.05, ^**^P < 0.01, ^***^P < 0.001 vs. CUMS group; by one-way ANOVA followed by Bonferroni post-hoc test

As shown in Fig. 4a–d hippocampal PCSK9 mRNA expression by PCR was significantly (*P* < 0.001) increased (F _(4, 25)_ = 20.86, *P* < 0.001), in rats experienced 6 weeks of CUMS compared to control group. same results were exhibited in the hippocampal TLR-4, NF-κB and IDO-1 mRNA (F_(4,25)_ = 79.22, *P* < 0.001, F_(4,25)_ = 91.77, *P* < 0.001, F_(4,25)_ = 93.91, *P* < 0.001, respectively) which were significantly increased, compared to control group Aliro-M and Alior-H led to a significant decrease in hippocampal PCSK9 and NF-κB mRNA expression (P < 0.01, P < 0.001 respectively) and both led to a significant*(P* < 0.001) decrease in TLR-4 and IDO-1 mRNA expression relative to CUMS group.

### Effect of Alirocumab on CUMS-Induced Changes in Hippocampal Tryptophan, Kynurenine Concentrations

As depicted in Fig. 4e, f CUMS induced a significant (*P* < 0.001) decrease in hippocampal tryptophan (F _(4, 25)_ = 13.88, *P* < 0.001) and significant increase in kynurenine concentrations (F _(4, 25)_ = 124.3, *P* < 0.001) compared to control group. Aliro-M and Aliro-H significantly increased hippocampal tryptophan (P < 0.01, P < 0.001) and significantly decreased kynurenine (P < 0.05, P < 0.001) concentrations respectively compared to CUMS group.

### Effect of Alirocumab on CUMS-Induced Changes in Hippocampal HMGB1 and RAGE, NLRP3 Inflammasome Complex and Mature IL-1β Protein Expression in Rats

**Fig. 5:**
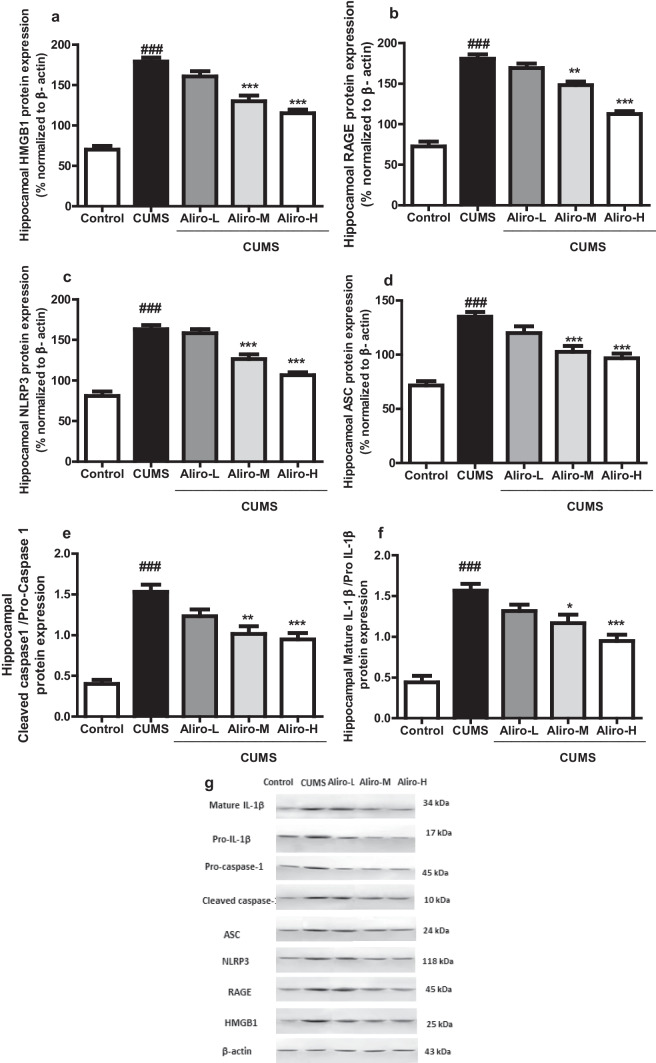
Effect of alirocumab (Aliro-L,Aliro-M and Aliro-H) on CUMS-induced Changes in Hippocampal HMGB1, RAGE, NLRP3 inflammasome complex and mature IL-1β protein expression; **a** HMGB1, **b** RAGE, **c** NLRP3, **d **ASC, **e** Cleaved Caspase-1/Pro-caspase-1 and **f** MatureIL-1β/pro-IL-1 β **g** a representative image and quantitative protein levels were normalized to β-actin. ^###^*P* < 0.001 vs. control group and^*^P < 0.05, ^**^P < 0.01, ^***^P < 0.001 vs. CUMS group; by one-way ANOVA followed by Bonferroni post-hoc test

As shown in Fig. 5 a significant increase in the hippocampal HMGB1 (F _(4,25)_ = 55.10, *P* < 0.001), RAGE (F _(4,25)_ = 75.43, *P* < 0.001), NLRP3 (F _(4,25)_ = 47.78, *P* < 0.001), apoptosis-associated speck-like protein (ASC) (F _(4,25)_ = 23.40, *P* < 0.001), Cleaved Caspase-1/ Pro-caspase-1 (F _(4,25)_ = 26.85, *P* < 0.001) and mature IL-1β/pro-IL-1β (F _(4,25)_ = 24.65, *P* < 0.001) protein expressions in CUMS group compared to control group. Both Aliro-M and Aliro-H significantly (*P* < 0.001) reduced the hippocampal HMGB1, NLRP3 and ASC protein expressions and significantly reduced RAGE (*P* < 0.01, *P* < 0.001 respectively), Cleaved caspase-1/pro-caspase-1 (*P* < 0.01, *P* < 0.001 respectively) and mature IL-1β/pro-IL-1β (*P* < 0.05, *P* < 0.001 respectively) protein expression compared to CUMS group.

### The Correlation of Hippocampal IL-1β, IL2, IL-6, TNF-α, NF-κB, RAGE, HMBG1, IDO-1, Kynurenine, Tryptophan, NLRP3 Inflammasome Complex and PCSK9 Level

A positive correlation was exhibited in all tested groups between the hippocampal PSCK9 and the hippocampal IL-1β, IL-2, IL-6, TNF-α, NF-κB and the kynurenine concentration as well as HMBG1, RAGE, NLRP3 inflammasome complex protein expressions. A negative correlation was found between tryptophan concentration and hippocampal PCSK9 (Table 1).

**Table 1:** The correlation among hippocampal IL-1β, IL2, IL-6, TNF-α, NF-κB, RAGE, HMBG1, IDO-1, Kynurenine, Tryptophan, NLRP3 inflammasome complex and PCSK9

	IL-1β		IL-2		IL-6		TNF-α	
Correlation	r	P	r	P	r	P	r	P
PCSK9	0.63	0.0002	0.73	< 0.0001	0.75	< 0.0001	0.77	< 0.0001
	NF-κB		RAGE		HMGB1		IDO-1	
Correlation	r	P	r	P	r	P	r	P
PCSK9	0.81	< 0.0001	0.85	< 0.0001	0. 85	< 0.0001	0.80	< 0.0001
	Kyneurenine		Tryptophan		NLRP3		ASC	
Correlation	r	P	r	p	r	P	r	p
PCSK9	0.78	< 0.0001	-0.58	0.0008	0.84	< 0.0001	0.96	< 0.0001
	Caspase-1p20							
Correlation	r	P						
PCSK9	0.80	< 0.0001						

## Discussion

In the present study, CUMS protocol was used to induce depressive-like-behaviors in rats to assess the ability of PCSK9-inhibition by alirocumab, and its anti-inflammatory potentials, to ameliorate the induced depressive-like-behaviors and neuro-inflammation. Alirocumab was able to reduce neuro-inflammation which was exhibited by the reduction of hippocampal HMGB1, RAGE and TLR4 with subsequent decrease of inflammatory mediators; NF-κβ, IL-1β, IL-2, IL-6, TNF-α in addition to NLRP3 inflammasome complex. Alirocumab was also able to restore the balance between kynurenine and tryptophan concentrations by down-regulation of hippocampal IDO-1. These effects were favorably reflected on the depressive-like-behaviors in rats induced by the CUMS protocol.

CUMS is considered a reliable animal model for depression and the chronic unpredictable exposure to different stressors used in this protocol is a widely accepted method for induction of depression. It is well documented to recapitulate the main behavioral disturbances exhibited in human depression with high face, constructive and predictive validities. These potentials allow it to be used in studies exploring the molecular pathways involved in depression pathophysiology and assessing the anti-depressant impact of different drugs on these pathways**.** Results of different studies were in accordance to our results, where the use of CUMS protocol in rats led to lethargy and behavioral despair in OFT and FST respectively along with hyperactivation of hypothalamic pituitary axis (Willner 1997; Liu et al. 2016; Abuelezz et al. 2017; Pesarico et al. 2021). These deleterious changes were restored in a dose dependent manner by Alirocumab.

More than 50 years ago, the hypothesis of reduced serotonin in depression was introduced as a possible pathological background mechanism for depression. It was hypothesized that the dysregulated serotoninergic system, implicating low levels of serotonin is a result of the switch of tryptophan metabolism from serotonin synthesis to kynurenine production. Normally, a small amount of tryptophan (~ 5%) is converted to serotonin, while the rest (~ 95%) is metabolized via the kynurenine pathway. So, even a minor change in the activity of the kynurenine pathway was found to have a significant impact on the tryptophan pool in the brain. Tryptophan is metabolized to kynurenine by IDO-1 which represents the rate‑limiting enzyme for this pathway. IDO-1 is induced by different pro-inflammatory cytokines including IL-1β, IL-2, IL-6, and TNF-α. Accordingly, studying the pathological underpinnings of depression has highlightened the important role of inflammatory pathways in driving changes in neuronal regulation of tryptophan/kynurenine pathway (Anderson and Maes 2014; Wang et al. 2019; Zádor et al. 2021).

On the other hand, HMGB1 is a ubiquitously expressed chromatin intracellular protein which is involved in triggering and amplifying inflammatory processes. In brain, HMGB1 has emerged as one of the key players in the pathophysiology of neuro-inflammation. It exerts its pathological effects through binding to RAGE and TLR4 leading to neuro-inflammation by enhancing pro-inflammatory cytokine (TNF-α, IL-1β, IL-2 and IL-6) synthesis through activation of the NF-κB signaling pathway. (Zhang et al. 2019a; Zádor et al. 2021). NF-κB was also found to be involved in the upregulation of NLRP3 inflammasome. The inflammasomes, are intracellular polyprotein complex which consist of NLRP, ASC and caspase-1. They represent a part of the intracellular sensors of danger- or damage-associated molecular patterns (DAMPs). Inflammasomes are now recognized as crucial mediators in inflammation. Although numerous inflammasomes have been identified, such as NLRP1, NLRP2, NLRP3, yet NLRP3 inflammasome represents the most extensively studied and the best understood member in the NLRP family. Activation of NLRP3 inflammasome was linked to the pathophysiology of different cardiovascular diseases and neurological diseases including depression by promoting the cleavage of pro-IL-1β to produce mature IL-1β. NLRP3 inflammasome via IL-1β was found to induce PCSK9 secretion. The first step in NLRP3 inflammasome activation appears to occur through an initiating signal in which DAMPs are recognized by TLRs, leading to activation of NF-κB-mediated signaling, which in turn up-regulates transcription of inflammasome-related components (Shao et al. 2015; Zhang et al. 2019b; Ding et al. 2020; Tong et al. 2020; Wang et al. 2020; Xu et al. 2020). Accordingly, a new strategy for prevention and treatment of depression has emerged targeting inflammation (the pro-inflammatory cytokines and the pathways involved in their upregulation and overactivation) to ameliorate signs of depression through reducing IDO-1.

Results of our study demonstrated that rats exposed to CUMS protocol exhibited increased levels of IDO and kynurenine and decreased levels of tryptophan compared to the control group. These results are in line with results of several previous studies (Abuelezz et al. 2017; Zhang et al. 2019b). Aliro-M and Aliro-L concurrent treatment of animals significantly counteracted these deleterious effects and restores the balance of the tryptophan/kynurenine which shifts the tryptophan pool towards the serotonin pathway by down-regulation of hippocampal IDO-1.

Our study confirmed the inflammatory state present in the CUMS group exhibited in the form of elevated hippocampal levels of IL-1β, IL-2, IL-6, and TNF-α in addition to overactivation of HMGB1/RAGE/TLR4 axis and NLRP3 inflammasome compared with the control group. These results are in accordance with results of different earlier studies (Abuelezz et al. 2017; Zhang et al. 2019a, b; Wang et al. 2020; Xu et al. 2020). The anti-inflammatory effect of the PCSK9 inhibitor was exhibited as evident by Aliro-M and Aliro-L ability to significantly decrease the elevated hippocampal inflammatory cytokines, HMGB1/RAGE/TLR4 axis and NLRP3 inflammasome and these effects were correlated with PCSK9 expression. The involvement of PCSK9 in depression was highlightened with genetic analyses studies exhibiting a positive association between increased risk of depressive mood and circulating levels of PCSK9. Also its contribution in inflammation was documented in different previous studies, and it was claimed that HMGB1/RAGE/TLR4 with subsequent NF-κB activation and the inter-twined relationship between NLRP3 inflammasome, IL-1β and PCSK9 may be the key signaling pathways that account for PCSK9-induced inflammatory response with subsequent release of the different inflammatory mediators (Apaijai et al. 2019; Ding et al. 2020; Lei et al. 2020; Abuelezz and Hendawy 2021; Macchi et al. 2021).

Finally, our results exhibited that alirocumab by its ability to suppress PCSK9 exerts anti-inflammatory potentials that can alleviate depressive-like behaviors through reducing hippocampal IDO-1 and restoring the balance between tryptophan and kynurenine pools. Although this ability to suppress PCSK9 by alirocumab was positively correlated to its ability to decrease the HMGB1/RAGE/TLR4 axis and NLP3 inflammasome, however, further studies are required to explore more and confirm the effect of alirocumab on different neurotransmitters involved in depression as serotonin and other downstream mediators of those pathological pathways and correlate the antidepressant effect to the right specific mediators. Thus, our results could be translated in the future clinically to patients using Alirocumab for the control of dyslipidemia who might gain more benefits by reducing the associated long term depressive complications which is known to be increased in cardiovascular patients. Accordingly, PCSK9-inhibitors could act as a double weapon for both conditions in these patients. Nonetheless, more experimental and clinical studies are recommended in this context.

## Data Availability

All data generated or analysed during this study are included in this published article.
